# Self-efficacy beliefs of medical students: a critical review

**DOI:** 10.1007/s40037-018-0411-3

**Published:** 2018-02-26

**Authors:** Robert M. Klassen, Joel R. L. Klassen

**Affiliations:** 1 University of York, York, UK; 2 Hull York Medical School, York, UK

**Keywords:** Medical students, Self-efficacy, Motivation, Medical education

## Abstract

**Introduction:**

Self-efficacy is a theoretically and empirically robust motivation belief that has been shown to play an important role in the learning and development of new skills and knowledge. In this article, we critically review research on the self-efficacy beliefs of medical students, with a goal to evaluate the existing research and to strengthen future work. In particular, we sought to describe the state of research on medical student self-efficacy and to critically examine the conceptualization and measurement of the construct. Finally, we aimed to provide directions for future self-efficacy research.

**Methods:**

We critically reviewed 74 published articles that included measures of self-efficacy beliefs of medical students.

**Results:**

Our review showed that (a) research on the self-efficacy beliefs of medical students is growing and is becoming increasingly international, and (b) that nearly half (46%) of self-efficacy measures showed conceptual and operational flaws.

**Discussion:**

Our critical review of 74 research studies on self-efficacy of medical students found that although research in the field is increasing, nearly half of measures labelled as self-efficacy were incongruent with the conceptual guidelines set by self-efficacy experts. We provide five suggestions for future research on the self-efficacy of medical students.

**Electronic supplementary material:**

The online version of this article (10.1007/s40037-018-0411-3) contains supplementary material, which is available to authorized users.

## What this paper adds

Self-efficacy beliefs facilitate the learning and development of medical students. Although research on the self-efficacy beliefs of medical students is of great interest in medical education, no attempts have been made to systematically review the research and to examine the validity of the measurement tools used in the research. The findings from this review suggest that research on medical student self-efficacy is growing rapidly and is becoming increasingly international, but that much research is not aligned with the conceptual underpinnings of the construct, thus reducing the validity of its measurement.

## Introduction

Medical educators are increasingly interested in the motivation beliefs of their students. In particular, interest is growing in how medical students’ self-efficacy is related to learning and development during medical school [1]. Bandura’s social cognitive theory suggests that self-efficacy—defined as the confidence to carry out the courses of action necessary to accomplish desired goals [2]—plays an important role in influencing achievement outcomes through its dynamic interplay with environmental and behavioural determinants [3]. Although skills and knowledge provide the raw materials for student success in medical education, beliefs about personal capabilities to use these raw materials can spell the difference between success and failure.

Self-efficacy beliefs provide the underpinning for motivation, well-being, and achievement and ‘are rooted in the core belief that one has the power to effect changes by one’s actions’ (P. 622) [4]. According to self-efficacy theory [2], the factors that influence behaviour are embedded in the belief that one has the capability to accomplish that behaviour. In most cases, people will choose to engage in activities in which they are confident of success, and avoid those in which they are not. This is particularly critical in intense learning environments such as medical school, where learning is dependent on overcoming a range of doubt-inducing intellectual, social, and motivational challenges. Research on the self-efficacy beliefs of medical students builds understanding of students’ choices, level of effort, and persistence, and has the potential to inform instructional practices [1].

Self-efficacy is an important motivational factor for the development of medical students, but few attempts have been made to systematically document the growth and focus of research in the area, or to critically examine if the measurement and conceptual problems that have hampered self-efficacy research in other fields are also found in research on medical students. The current critical review is not intended to summarize the substantive findings of this body of research, but rather aims to:

- provide a description of self-efficacy research involving medical students, with attention paid to growth in research quantity and international reach of the research;
- critically evaluate the conceptual fidelity of measurement of medical student self-efficacy;
- propose directions for future research on the self-efficacy beliefs of medical students.

### Self-efficacy and its relation to other constructs

The robustness of research on self-efficacy depends on valid assessment of its key constructs. In Bandura’s social cognitive theory of human agency [2], self-efficacy reflects internal personal beliefs that interact bi-directionally with behavioural and environmental determinants, illustrated in a model of triadic reciprocal causation. Self-efficacy operates as an intra-personal motivation variable that captures the core aspects of human agency, namely people’s beliefs that they are contributors, but not sole determiners, of what happens to them.

According to Bandura’s conceptualization, self-efficacy is characterized by: (a) beliefs about future actions, not past performance; (b) beliefs about capabilities, not outcome expectations; and (c) domain specificity, not assessment of generalized traits [1]. Other constructs bear conceptual similarity to self-efficacy. For example, self-efficacy is conceptually separable from *confidence*. Although the two constructs are sometimes used interchangeably by researchers, confidence is a ‘catchword rather than a construct embedded in a theoretical system’ (P. 382) [2]. Self-confidence has been the attention of research but with a relatively modest conceptual foundation. Self-confidence reflects strength of belief (*She is a self-confident person*), but not the target or specific domain for that belief. Research on self-efficacy offers the advantage of building on a strong theoretical foundation that provides a deeper understanding of human agency.

Self-efficacy is separable from other constructs such as *self-concept*, which refers to multidimensional self-perceptions that are past-oriented, aggregated, and normative; *self-esteem*, which refers to personal judgments of self-worth; or *locus of control*, which refers to generalized beliefs that actions affect outcomes [5]. In contrast, self-efficacy beliefs are goal-oriented, context specific, and future-oriented judgments of capabilities that change according to the task involved [6]. Self-efficacy refers to beliefs about *capabilities* rather than evaluation of past success or judgments about outcome expectations that flow from self-efficacy [7]. Operationally, self-efficacy measures typically include words indicating assessment of capability, such as *can* and *confident*: ‘I am confident that I can solve this problem.’ Finally, self-efficacy is domain-specific, not a generalized trait of self-confidence that does not specify a particular task or domain [7]. People differ in their efficacy across different domains of functioning; the construction of valid self-efficacy scales requires attention to specific domains of functioning, rather than overall well-being [3].

### Self-efficacy of medical students

Medical educators benefit from building their understanding of why some students excel and others struggle during medical training [8]. Thus, we considered it suitable to explore the body of research that examines a well-studied motivation force—self-efficacy—in medical students. Our review of the literature reflects an increasing awareness in medical education that self-efficacy plays an important role in student learning and development, but also that the field lacks an appraisal of recent research that might signpost profitable future directions.

## Methods

In this critical review we focused on the self-efficacy beliefs of students in undergraduate medical education and so did not include literature involving specialty or professional training. A ‘critical’ review serves two functions: it provides a description of research conducted with a particular focus and it provides a critical appraisal—a careful and systematic examination designed to judge its trustworthiness and value—of that research [9–11]. The search was restricted to English-language peer-reviewed journal articles found on PsycINFO, MEDLINE and Embase for literature that was published between 1989 (the year of the publication of Bandura’s seminal *Human agency in social cognitive theory *[12]) through to May 2016. The search combined the index term ‘medical student’ with keywords (*medical student *or *medical education*) AND *self-efficacy*.

We included the term ‘self-efficacy’ but chose to exclude studies on related constructs—*confidence, self-confidence,* and *self-perceived competence*—since self-efficacy has a distinct well-developed theoretical foundation and empirical research base, whereas related constructs such as self-confidence may lack this foundation [2]. We chose to exclude book chapters, theses, dissertations, and conference presentations, in an attempt to include literature with a relatively consistent and standard peer-review process. The articles resulting from this search (*n* = 784) were hand-searched by one author who removed papers that were not relevant by reading the abstracts. Full-text versions of the remaining identified articles (*n* = 157) were subsequently obtained where possible for a more detailed assessment.

The resulting articles were read to determine if the article: (a) reported one or more empirical studies (not systematic qualitative or quantitative reviews, or theoretical articles), (b) reported a measure labelled as ‘self-efficacy,’ and (c) included participants who were undergraduate students enrolled in a medical school. After hand-searching the 157 articles, 76 articles did not meet the study criteria, and 7 studies were not available (no download or inter-library loan available; no response after author contact), leaving 74 studies to be reviewed for this study.

We recorded study characteristics including year of publication, methodology, geographical location of researchers’ affiliation, sample attributes (sample size, number of universities represented in sample), journal name, and domain of research focus. In addition, we systematically compared the congruence of measures used in the reviewed studies with the measurement guidance provided by Bandura and other prominent self-efficacy researchers and theorists [3, 5, 7, 13]. Based on this guidance, we evaluated three aspects of the measures labelled as ‘self-efficacy’:

- Is the measure future oriented (not an evaluation of past performance or current skill level)?
- Does the measure focus on beliefs about capability to carry out the courses of action necessary for success (and not outcome expectations or intentions to act)?
- Does the measure focus on a particular domain (i. e., not general self-confidence)?

## Results

### Description of reviewed studies

**Fig. 1:**
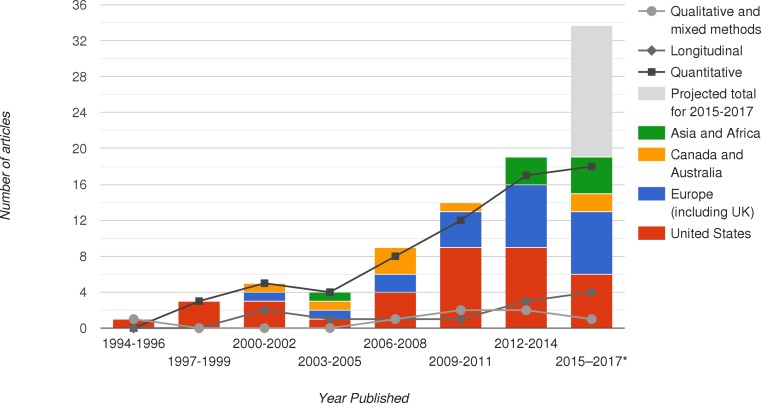
Summary of research on medical student self-efficacy: publication rates, research internationalization. *Note Articles were reported only for five months in 2016

We retrieved 74 empirical articles that measured the self-efficacy beliefs of medical students. Articles were published in 36 separate journals, with highest frequency of publications in *Advances in Health Sciences Education* (*n* = 7) and *BMC Medical Education* (*n* = 7). Fig. 1 presents a breakdown of the studies by 3‑year period, by geographical region, and by methodology. As seen in the figure, the number of publications focused on medical students’ self-efficacy is increasing, with 1 article published between 1994 and 1996, increasing to 19 articles published in the last 17-month period covered in the review (i. e., 2015 until May 2016), with a projected total of over 30 articles for the 3‑year period 2015–2017. Research affiliations have become increasingly international over time, with the early studies conducted by researchers at American universities (i. e., from 1994–1999), with an increasing number of non-US affiliated researchers over time. Researchers from Asia and Africa were weakly represented from 1994–2011, with growing representation over the last five years. Only 3 countries were represented between 1994–2002, 7 countries between 2003–2010, and 15 countries represented post-2010. Sample sizes within each study ranged from 12 to 1646, with a mean sample size of 256.

### Research design

Most studies (68/74, 92%) used a quantitative research design with questionnaires assessing level of self-efficacy beliefs, with 6 studies (8%) using mixed methods, and 1 study [14] using a qualitative design. Most studies (63/74, 85%) used a cross-sectional design, with 10 studies (13.5%) using a pre-post or 2‑wave longitudinal design, and 2 studies (3%) using longitudinal designs with three or more waves [15]. Fifty-nine of the studies (80%) collected data from samples at a single site (university, medical school, or health centre) and 15 studies included data from multiple sites (range: 2–34 sites).

### Substantive focus of articles

Self-efficacy is hypothesized to influence behaviours and environments, and in turn to be influenced by them [2]. We found that researchers used self-efficacy both as a predictor variable (e.g., *Is anatomical self-efficacy related to anatomy assessment scores*) [16]? and as an outcome variable (e.g., *Did surgical self-efficacy increase after exposure to cognitive task analysis curriculum*?) [17]. Most studies with self-efficacy as outcome variable showed that curriculum interventions boosted self-efficacy alongside assessment scores. Four studies reported self-efficacy scale validations, with scales developed with the purpose of assessing self-efficacy for medical skills [1], for palliative care [18], for effective practice [19], and for developing a patient-centred focus [20].

### Measurement issues

Measurement problems were common in the reviewed studies, with almost half of the reviewed studies using measures incongruent with theory and guidelines provided for scale construction [4]. Tab. 1 displays summarized results from the analysis of the theoretical congruence of self-efficacy measures (a comprehensive table of results [Table S1] is provided as Online Electronic Supplementary Material). In Tab. 1, examples are first given of measures that show congruence with theory in terms of their conceptualization and domain specificity. Next, we provide examples of measures that are not congruent with theory due to: absence of future orientation (*examples a and b*), measurement of outcome expectancies, not perceived capabilities (*example c*), measurement of alternative constructs, including self-esteem and anxiety, (*examples d–f*), measurement of breadth of medical education (*example* g), and measurement of external challenges, not personal capabilities (*example h*).

**Table 1:** Congruence of self-efficacy measurement with theory

	*n* = 68^a^	Key features	Examples
Congruent with theory	37 (54%)	Conceptualization	*I am confident that I can handle the most difficult parts of the tasks during the simulator training*
		Domain specificity	*How confident are you that you can convey to your patients the information they need to quit smoking?*
Not congruent with theory	31 (46%)	Conceptualization	(a) *How would you rate your research skills?* (not future-oriented) (b) *I got plenty of opportunities to develop procedural skills *(not future-oriented) (c) *I expect to do well in this course* (measure of outcome expectancies, not perceived capabilities) (d) *I trust in my intellectual abilities* (measure of self-esteem) (e) *I believe my fellow students respect me *(self-esteem) (f) *I feel anxious about having patients with disabilities* (measure of anxiety) (g) *Geriatrics education was part of all four years of my medical education *(measure of breadth of medical training) (h) *Rural practice is too hard* (measure of external challenges, not personal capabilities)
		Domain specificity	(i) *I can always manage to solve difficult problems if I try hard enough *(general problem-solving, not perceived capabilities to carry out a particular task)

^a^Only 68 out of 74 total articles provided examples or clear descriptions of self-efficacy measures

Lack of domain specificity was noted in three studies that used Schwarzer’s General Self-Efficacy Scale [21], in which items do not specify a particular domain of capability (e.g., *I can always manage to solve difficult problems if I try hard enough, example i*). Overall, of the 68 (out of 74) studies that provided examples (or a clear description) of the content of measures, 37 (54%) used self-efficacy with conceptually congruent measures, with the remaining 31 studies (46%) using measures that are not congruent with guidelines derived from self-efficacy theory, and capturing a wide range of other constructs.

## Discussion

Self-efficacy is a key factor in human agency: people who lack confidence in the skills they possess are less likely to engage in tasks which require those skills, and are less likely to persevere when faced with obstacles and challenges [22]. The findings from this critical review show that research on the self-efficacy of medical students is increasing, with a growing number of researchers in a growing number of international contexts exploring how self-efficacy is associated with student learning and achievement. Continuing research is needed to explore the dynamic nature of self-efficacy in a range of medical school contexts, with a clear need for research that examines the contributing sources of self-efficacy.

### Future directions for self-efficacy research in medical education

#### Conceptual clarity and measurement fidelity

Problems with conceptual clarity and measurement fidelity were found in almost half of the studies reviewed. The pervasiveness of measurement problems creates a serious threat to the future of self-efficacy research in medical education. Mis-measurement and lack of attention to conceptual clarity results in uncertainty about findings, and a lack of progress in understanding the role self-efficacy plays in influencing motivation and academic performance. Problems with ambiguous and conceptually faulty self-efficacy measurement can be avoided by researchers who are committed to using measures congruent with established theory, and by reviewers who are vigilant in evaluating the quality of self-efficacy measures. Theoretical and operational challenges of self-efficacy theory and measurement are not to be discouraged in future research; however, atheoretical and ad hoc measures do little to advance our knowledge of how a theoretically and empirically robust construct operates in medical students. Research involving measures of other motivation constructs and self-beliefs (e.g., self-concept, self-esteem, expectancy outcomes) is to be encouraged, but valid measurement is a fundamental research principle; idiosyncratic operationalization of established constructs does not result in increased understanding of a phenomenon.

#### More sophisticated and varied designs

The results of our review show that most studies were cross-sectional, one-shot studies conducted in a single setting: only 20% of studies collected data from more than one site. Cross-contextual comparisons are useful in building theory and practical applications because they provide researchers with ‘a valuable heuristic basis to test the external validity and generalizability of their measures, theories, and models’ (P. 59) [23]. Cross-contextual research also provides insight into the relative self-efficacy beliefs of medical students under different kinds of training regimes (e.g., problem-based learning versus traditional programs).

Self-efficacy beliefs are dynamic and would be expected to change through students’ medical training. Researchers in our review identified the need for greater attention to longitudinal self-efficacy research [17]. Several studies used pre- and post-test (i. e., two-wave) designs to measure changes in self-efficacy, but true longitudinal designs require three or more waves of data to reliably establish patterns of change [24]. We urge researchers to design studies that trace the development of medical students’ self-efficacy beliefs over multiple (>2) time periods in order to better understand trajectories of students’ self-efficacy development through medical training. Finally, few studies used anything other than quantitative designs, with only one study using a qualitative design. Further studies that include the additional depth and richness associated with qualitative research approaches may provide useful insight into the self-efficacy beliefs of medical students.

#### Sources of self-efficacy

A logical next step for researchers is to work toward a clearer understanding of how medical students’ efficacy beliefs develop and take root during undergraduate training. Fortunately, some attention is being paid to the sources of self-efficacy in medical education [14, 25]. Despite these initial efforts, more research in this area is warranted. Bandura [2] contended that the relationship between the hypothesized sources of self-efficacy and self-efficacy beliefs varies as a function of contextual and social factors. For researchers interested in motivation interventions that target self-efficacy, attention to the sources of self-efficacy may provide a promising avenue for further work. People acquire self-efficacy beliefs based on the cognitive processing and interpretation of their enactive and vicarious experiences, verbal persuasions, and physiological reactions to stressful situations. These four sources do not automatically influence self-efficacy; rather, contextual and social factors influence how people interpret and act on the sources of self-efficacy [22]. In order to understand how self-efficacy develops in medical students, further work is needed to understand how students acquire and process information gained from the sources of self-efficacy.

## Limitations

Our decision to sample journal articles (not book chapters, conference presentations, or theses and dissertations) written in English undoubtedly restricts the capture of international research on medical student self-efficacy. We based our evaluation of the conceptual clarity and measurement fidelity of research based on the originator and adherents of self-efficacy theory, but other perspectives on ‘good’ measures may offer findings and interpretations that are opposed to those we espouse in this review. For example, Schwarzer’s [21] espousal of a general self-efficacy directly opposes Bandura’s conceptualization of domain-specific self-efficacy, and although Bandura and his adherents find fault with the notion of general self-efficacy [2], the perspective of Schwarzer on generalized self-efficacy should be acknolwedged and debated. We acknowledge that our stance is firmly in the Bandurian camp of self-efficacy research, and we believe that the empirical underpinning of the research conducted from this stance is robust and that the theoretical foundation is sound.

## Conclusions

The quantity of self-efficacy research in medical education has increased steadily over the last decades but questions remain about the quality of some of the research. Our critical review found that nearly half of the measures labelled as self-efficacy were incongruent with the conceptual guidelines proposed by self-efficacy experts. As recognition of the importance of self-efficacy of medical students continues to grow, it is important that researchers use measures that are aligned with the construct’s conceptual roots, in order to maximize explanatory value and predictive power. We are optimistic that research on the motivation beliefs—and especially self-efficacy—of medical students is worth pursuing, but we caution researchers to use care in designing future studies by following conceptual and methodological guidelines.

## Caption Electronic Supplementary Material

Table S1 Overview of 74 studies included in review
