# Supplementary material for: Self-efficacy beliefs of medical students: a critical review
Source: Perspect Med Educ. 2018 Feb 26;7(2):76–82. doi: 10.1007/s40037-018-0411-3 (PMC5889382; doi:10.1007/s40037-018-0411-3)
Supplement: Supplementary file 1 — Table S1 Overview of 74 studies included in review [file 40037_2018_411_MOESM1_ESM.docx]

**Table S1** Overview of 74 studies included in review

| **Authors** | **Title** | **Journal** | **Year** | **Self-efficacy related design** | **Participants** | **Country** | **SE-related findings** | **SE measure examples** | **Congruent with theory?** |
| --- | --- | --- | --- | --- | --- | --- | --- | --- | --- |
| Aboalshamat, Khalid; Hou, Xiang-Yu; Strodl, Esben | Psychological well-being status among medical and dental students in Makkah, Saudi Arabia: A cross-sectional study. | Medical Teacher | 2015 | Quant - Cross-sectional; General Self-Efficacy scale (GSE) (Schwarzer & Jerusalem 1995). 10 questions with a four-point (1–4) scale, providing sum score ranges from 4 to 40 | Years 2 and 3 at one university | Saudi Arabia | GSE not related to academic achievement | Not provided, but used GSE, e.g., 'I can always manage to solve difficult problems if I try hard enough' | Not domain specific; general self-efficacy |
| Aboumatar, H. J; Thompson, D; Wu, A; Dawson, P; Colbert, J; Marsteller, J; Kent, P; Lubomski, L. H; Paine, L; Pronovost, P | Development and evaluation of a 3-day patient safety curriculum to advance knowledge, self-efficacy and system thinking among medical students | BMJ Quality and Safety | 2012 | Quant- Cross-sectional; A survey containing 9 'I know how to' statements that med students rated on Likert-scales from 1-5. Items piloted in earlier safety and ward prep courses | 2nd year in 1 med school | USA | SE increased after 3-day patient safety session | SE was measured using nine 'I know how to' statements, e.g., 'I know how to investigate a defect' | Not future oriented; assesses current knowledge, not capability to carry out a course of action |
| Ahlborg L; Weurlander M; Hedman L; Nisel H; Lindqvist PG; Fellander-Tsai L; Enochsson L | Individualized feedback during simulated laparoscopic training:a mixed methods study. | International Journal of Medical Education | 2015 | Quant - Self-efficacy was self-assessed before and after the simulator training session using a 3-item questionnaire where each item was rated on a 7-grade Likert-type scale | 5th year from one university | Sweden | Overall self-efficacy scores improved with training in both groups. Males scored significantly higher than females on self-efficacy on some items | I am confident that I can handle the most difficult parts of the tasks during the simulator training/ future simulator training' | Congruent |
| Ajuwon GA | Computer and internet use by first year clinical and nursing students in a Nigerian teaching hospital | BMC Medical Informatics & Decision Making | 2003 | Quant - Cross-sectional questionnaire measuring self-efficacy for performing internet tasks | 1st year in one university | Nigeria | A total of 86.2% of medical students were "very confident" to download materials from the Internet compared to 13.8% of student nurses. Males had higher SE than females | Confidence in performing 5 internet-related tasks with responses of Very confident; little confident; not confident at all | Congruent: |
| Ammentorp, J; Thomsen, J. L; Jarbol, D. E; Holst, R; Ovrehus, A. L. H; Kofoed, P | Comparison of the medical studnets' perceived self-efficacy and the evaluation of the observers and patients | BMC Medical Education | 2013 | Quant - Cross-sectional; med students' confidence levels of successfully managing 12 communication skills assessed on a Likert scale | One university | Denmark | Students generally rated their (SE) performance on the CCOG checklist lower than observers or SPs. SE varied across tasks | How confident are you in successfully managing communication skills? | Congruent |
| Aper, L; Reniers, J; Koole, S; Valcke, M; Derese, A | Impact of three alternative consultation training formats on self-efficacy and consultation skills of medical students | Medical Teacher | 2012 | Quant - Pre-post quasi-experimental using a 9 item SE scale | 2nd year in one university | Belgium | One out of 3 training types boosted SE. SE was not boosted by traditional training (but did boost cognitive consultation performance scores. | How confident are you that you can perform a thorough physical exam?' | Congruent |
| Artino, A. R; Dong; DeZee, T; Gilliland, K. J; Waechter, W. R; Cruess, D. M; Durning, D. F | Development and initial validation of a survey to assess students' self-efficacy in medical school | Military Medicine | 2012 | Quant: scale validation of 19 item questionnaire | All med students in one university | USA | Scale validation. Students' SE increased from Year 1 to year 4. | How confident are you that you can… apply knowledge, etc.' | Congruent |
| Artino, A. R; Hemmer, P. A; Durning, S. J | Using Self-Regulated Learning Theory to Understand the Beliefs, Emotions, and Behaviors of Struggling Medical Students | Academic Medicine | 2011 | Quant - 5-item self-efficacy subscale assessed student confidence to learn the material presented in the course | 2nd year in one university | USA | Struggling students reported lower self-efficacy beliefs (d = .33). SE is context specific | How confident are you that you can... apply knowledge, etc. | Congruent |
| Artino, A. R; La Rochelle, J. S; Durning, S. J | Second-year medical students' motivational beliefs, emotions, and achievement | Medical Education | 2010 | Quant - (2-wave) Longitudinal study. SE measured in an online survey after 1st term of intervention. Survey composed of 26 items, employing 5-point, Likert style rating scales. | 2nd year in 1 med school | USA | SE was inversely related to anxiety, but SE was not significantly related to outcomes. | I'm confident that I can do an outstanding job on the activities in this course' | Congruent |
| Asgary, R; Saenger, P; Jophlin, L; Burnett, D. C. | Domestic global health: a curriculum teaching medical students to evaluate refugee asylum seekers and torture survivors. | Teaching & Learning in Medicine | 2013 | Quant - pre-post measures of general knowledge about physical and psychological sequelae of torture | 1st to 4th year med stds from one university | USA | SE increased in the post-test following training | I understand the psychological effects of torture' | Not future oriented; not focused on capabilities |
| Ault GT; Sullivan M; Chalabian J;. Skinner KA | A focused breast skills workshop improves the clinical skills of medical students. | Journal of Surgical Research | 2002 | Quant - A five-item pre- and postclerkship self-efficacy rating scale. The rating scale asked the students to self-rate their skills in the areas of physical examination, mammogram and ultrasound interpretation, lump detection, and the ability to teach breast self-examination, as well as problem-solving and management issues | 3rd year from 1 university | USA | The students who participated in the workshop demonstrated significantly higher self-efficacy rating change in their breast examination skills at the end of the clerkship compared to the control group | Self-rate skills in physical examination | Not future oriented or focused on beliefs about capabilities. SE operationalized as self-ratings of skills |
| Bierer, Prayson, & Dannefer | Association of research self-efficacy with medical student career interests, specialization, and scholarship: a case study | Advances in Health Sciences Education. | 2015 | Quant - cross-sectional comparison of clinical research SE of graduatess and matriculates | Multiple cohorts from 1 university | USA | Graduates rated their clinical research SE higher than matriculates on SE scales | Ratings of confidence to perform research-related tasks and activities | Congruent |
| Black, ML; Curran, MC; Golshan, S; Daly, R; Depp, C; Kelly, C; Jeste, DV | Summer Research Training for Medical Students: Impact on Research Self-Efficacy | Clinical and Translational Science | 2013 | Quant - pre-post data collecation around summer research training | Students who had completed 1 year of medical school from many different unis | USA | Research self-efficacy increased significantly after completing the summer research training. Found no gender differences in reported self-efficacy. | I understand primary research methodology principles' | Focus on current skill level rather than focus on beliefs to carry out actions |
| Bosse, H. M; Schultz, J-H; Junger, J; Huwendiek, S; Nikendei, C | The effect of using standardized patients or peer role play on ratings of undergraduate communication training: A randomized controlled trial | Patient Education and Counseling | 2012 | Quant - RCT with medical students assigned to three training conditions. Questionnaires pre and post interventions. | 5th year from 1 university | Germany | SE increased in training with SPs and peer role-play, but SE increased more with peer role-play. | Please rate your overall competence in medical interviewing. How important is the medical interview? (How confident are you in your ability…) | Measured ccompetence and current skill level |
| Braeckman, L; De Clercq, B; Janssens, H; Gehanno, J. F; Bulat, P; Pauncu, E. A; Smits, P; van Dijk, F; Vanderlinde, R; Valcke, M | Development and evaluation of a new occupational medicine teaching module to advance self-efficacy and knowledge among medical students | Journal of Occupational & Environmental Medicine | 2013 | Quant - three wave longitudinal assessments of self-efficacy and knowledge | 3rd year from 1 university | Belgium | Self-efficacy rating at T3 had no significant impact on knowledge examination scores. SE scores increased with the intervention (new teaching module). SE was not associated with the knowledge score. | Students were asked to indicate the degree of certainty that they had of knowledge/mastery of subject matter | Measured knowledge, not capability to carry out course of action |
| Brock, D;. Abu-Rish, E; Chiu, C.-R; Hammer, D; Wilson, S; Vorvick, L; Blondon, K; Schaad, D; Liner, D; Zierler, B | Interprofessional education in team communication: Working together to improve patient safety. | BMJ Quality and Safety | 2013 | Quant - Pre- and post-measurements of Attitudes, Motivation, Utility and Self-Efficacy (AMUSE) | 4th year in 1 university | USA | Medical students reported higher postlevels of self-efficacy than did nursing (mean=3.67, SD=0.43) or pharmacy students (mean=3.56, SD=0.73) | Not provided | Not possible to evaluate |
| Burgoon, J. M; Meece, J. L; Granger, N. A | Self-efficacy's influence on student academic achievement in the medical anatomy curriculum | Anatomican Sciences Education | 2012 | Quant - investigating predictive nature of SE for academic performance. Surveys containing same self-efficacy measures were completed by first-year medical students after each of 4 gross anatomy assessments | 1st year from 1 university | USA | Anatomical SE predicted 2 of 4 exam scores. | I am confident that I can make the proper cuts in the cadaver as outline in the lab manual' | Congruent |
| Campbell, J; Tirapelle, L; Yates, K; Clark, R; Inaba, K; Green, D; Plurad, D; Lam, L; Tang, A; Cestero, R; Sullivan, M | The Effectiveness of a Cognitive Task Analysis Informed Curriculum to Increase Self-Efficacy and Improve Performance for an Open Cricothyrotomy | Journal of Surgical Education | 2011 | Quant - experimental study about learning open cricothyrotomy procedure | 3rd year from 1 university | USA | Cognitive task analysis curriculum increased surgical perf and SE compared to control group | Rate your confidence to: 'make the necessary incisions' | Congruent |
| Campos-Sanchez, A; Lopez-Nunez, J.A; Carriel, V; Martin-Piedra, M.A; Sola, T; Alaminos, M. | Motivational component profiles in university students learning histology: a comparative study between genders and different health science curricula. | BMC medical education | 2014 | Quant - cross-sectional study of SE for learning histology | 1st year in one university | Spain | Medical students reported higher SE than dentistry and pharmacy students | "I believe I can earn a grade of “A” in histology"... I am confident I will do well... | Congruent |
| Carson, J. S; Gillham, B; Kirk, L. M; Reddy, S. T; Battles, J. B | Enhancing Self-Efficacy and Patient Care with Cardiovascular Nutrition Education | American Journal of Preventive Medicine | 2002 | Quant - cardiovascular nutrition education intervention study with SE measures administered before and after educational intervention | 4th year from 1 university | USA | Cardio nutrition curriculum boosted SE compared to control | I am able to advise a patient on how to lower the saturated fat in his diet' | Focus on current skill level rather than focus on beliefs to carry out actions |
| Chaput de Saintonge, D. M; Dunn, D. M | Gender and achievement in clinical medical students: a path analysis | Medical Education | 2001 | Quant - longitudinal multi-item scale questions measuring perceived ability to reach learning goals | 3rd year from 1 university | UK | SE was similar for M and F. For F, SE was unrelated to exam results at any stage. Men's SE was not influenced by learning environment; womens’ was. | No example: they cite Bandura & Wood 1989 | Not possible to evaluate |
| Cleary, TJ; Dong, T; Artino, AR | Examining shifts in medical students' microanalytic motivation beliefs and regulatory processes during a diagnostic reasoning task | Advances in Health Sciences Education. | 2014 | Quant - A single-item measure was created to examine the participants’ confidence about their ability to generate the correct diagnosis at three separate times during the clinical reasoning course | 2nd year in one university | USA | The results indicated that the participants showed a medium decrease in their self-efficacy after receiving negative corrective feedback following the first iteration of the task, and an even larger decrease following the second round of corrective feedback | Confidence in ability to generate correct diagnosis (based on Bandura's 2006 guidelines) | Congruent |
| Cox, ED; Koscik, RL; Olson, CA; Behrmann, AT; McIntosh, GC; Kokotailo, PK | Clinical Skills and Self-Efficacy After a Curriculum on Care for the Underserved. | American Journal of Preventive Medicine | 2008 | Quant - Assessed changes in self-efficacy associated with three curriculum formats | 3rd years from 8 unis | USA | Students who received the established (readings only) curriculum demonstrated the only large decline in self-efficacy, specifically around their ability to make a difference in the lives of underserved patients. | I am comfortable working with an interpreter; Confident in my knowledge of community resources' | Focused on 'comfort' or past experience (some items closer to SE conceptualization) |
| Creutzfeldt, J; Hedman, L; Medin, C; Heinrichs, WL; Fellander-Tsai, L | Exploring virtual worlds for scenario-based repeated team training of cardiopulmonary resuscitation in medical students | Journal of medical Internet research | 2010 | Quant - longitudinal measure of SE during virtual reality game | 1st year in one university | Sweden | Self-efficacy increased from before to after each of the two training sessions. Lower in females | Not provided | Not possible to evaluate |
| Day, FC; Srinivasan, M; Der-Martirosian, C; Griffin, E; Hoffman, JR; Wilkes, MS | A comparison of Web-based and small-group palliative and end-of-life care curricula: a quasi-randomized controlled study at one institution | Academic Medicine | 2015 | Quant - quasi-randomized controlled trial with two conditions | 3rd year 1 university | USA | After participation, student self-efficacy improved in both groups in all three skills domains | “How confident are you about your skills in this area?” (“not” confident = 1 to “extremely” confident = 4) | Congruent |
| Demiroren, Turan, Oztuna | Medical students' self-efficacy in problem-based learning and its relationship with self-regulated learning | Medical Education Online | 2016 | Quant - cross-sectional questionnaires | 2nd and 3rd year students at 1 university | Turkey | SE increased as self-regulated learning increased | Not provided | Not possible to evaluate |
| Engel, S. S; Crandall, J; Basch, C. E; Zybert, P; Wylie-Rosett, J | Computer-Assisted Diabetes Nutrition Education Increases Knowledge and Self-Efficacy of Medical Students | The Diabetes Educator | 1997 | Quant - pre-post design using 8 item scale measuring self-efficacy, 5-point Likert scale used | 3rd year 1 university | USA | SE increased after computer-aided instruction | Not provided | Not possible to evaluate |
| Gerlach, C; Mai, S; Schmidtmann, I; Massen, C; Reinholz, U; Laufenberg-Feldmann, R; Weber, M. | Does interdisciplinary and multiprofessional undergraduate education increase students' self-confidence and knowledge toward palliative care? Evaluation of an undergraduate curriculum design for palliative care at a german academic hospital. | Journal of Palliative Medicine | 2015 | Quant - Pre-post survey design to evaluate students’ attitude toward palliative care (PC) and their estimation of self-efficacy | 5th year from one university | Germany | Overall, confidence as measured by the total score was considerably higher after the course than before the course among the students who provided two questionnaires that could be matched. | Participants asked to estimate their confidence in handling terminally ill and dying patients (very low confidence to very confident) | Congruent |
| Grant, A; Kinnersley, P; Field, M. | Learning contexts at two UK medical schools: a comparative study using mixed methods. | BMC research notes | 2012 | Mixed methods. The self-efficacy in self-directed learning scale was adapted to measure self-directedness of learning in medical students. | Final year from two unis | UK | Students in problem-based curricula are more likely to be self-directed in their learning, to have a more hol- istic approach to learning and a greater sense of self- efficacy. | Self-efficacy in self-directed learning scale, e.g., 'How well can you participate in class discussions?' | Congruent |
| Guntern, S., Korpershoek, H., & van der Werf, G. | Benefits of personality characteristics and self-efficacy in the perceived academic achievement of medical students | Educational Psychology | 2016 | Quant - cross-sectional study using SE questionnaire with 16 items | 1st and 2nd year students from multiple unis | Switzerland and Austria | SE predicted exam scores | I trust in my intellectual abilities' | Not future oriented; not focused on beliefs of capabilities |
| Ilic, D; Nordin, RB; Glasziou, P; Tilson, JK; Villanueva, E | A randomised controlled trial of a blended learning education intervention for teaching evidence-based medicine. | BMC Medical Education | 2015 | Quant - RCT assessing student self-efficacy assessed using the Evidence-Based Practice Question (EBPQ). | Undergraduate entry/graduate entry/international (3 universities, different years) | Australia | Perceived self-efficacy, attitudes and behaviour toward EBM was significantly higher in students who received the blended learning approach | "How would you rate your research skills?" | Not future oriented; not focused on beliefs of capabilities |
| Isaac, V; Walters, L; McLachlan, CS | Association between self-efficacy, career interest and rural career intent in Australian medical students with rural clinical school experience. | BMJ Open | 2015 | Quant - Cross-sectional study of medical students in 17 universities | Completed their RCS term (year unclear?) in 17 universities | Australia | Rural self-efficacy was associated with gender, that is, female students had higher self-efficacy compared to male students. Rural background is associated with higher rural self-efficacy | Rural practice is too hard; People tell me I should work in a rural setting' | Not future oriented; not focused on beliefs of capabilities |
| Jang, HW; Kim, KJ | Use of online clinical videos for clinical skills training for medical students: benefits and challenges. | BMC medical education | 2014 | Mixed methods. SE questionnaire comprised ten items which ask the participants to indicate their attitudes toward OSCE that are known to be associated with their performance in OSCE – i.e., self-efficacy | 3rd years and 4th years from 34 unis | Korea | OSCE. The number of OSCE videos that the students viewed was moderately associated with their self-efficacy and preparedness for OSCE | None provided, but based on Mavis (2001), e.g., 'Rate your confidence in your ability to conduct an interview…' | Congruent |
| Kahan, M; Wilson, L; Midmer, D; Borsoi, D; Martin, D. | Randomized controlled trial on the effects of a skills-based workshop on medical students' management of problem drinking and alcohol dependence. | Substance Abuse | 2003 | Quant - RCT with students placed in two workshop conditions. Four months after the OSCE students were sent a survey on their knowledge, attitudes, and behavior towards patients with alcohol problems. | 3rd years and 4th years from 5 unis | Canada | The follow-up survey found that the alcohol group had a sustained increase in self-efficacy in working with alcohol patients | None provided, but described as 'comfort level and confidence in clinical skills' | Mixed measure with assessment of 'comfort level' (and confidence) in clinical skills |
| Kaufman, D. M; Laidlow, T. A; Langille, D; MacLeod, H; Sargeant, J | Differences in Medical Students' Attitudes and Self-efficacy Regarding Patient-Doctor Communication | Academic Medicine | 2001 | Quant - cross-sectional questionnaire, 5 point Likert scale | 1st, 2nd, and 4th year in one University | Canada | SE for communication dropped from 1st to 2nd year and then increased in 4th year (but cross-sectional study). 'SE was highest for the entering class' | Described as 'judgment of their ability to deal with different doctor-patient situations… in terms of what to do, investment of effort, persistence, and level of anxiety' | Not congruent; not future oriented, not focused on beliefs of capabilities |
| Khan, A. S; Canserver, Z; Ausar, U. Z; Acemoglu, H | Perceived self-efficacy and academic performance of medical students at Ataturk University, Turkey | Journal of the College of Physicians & Surgeons- Pakistan | 2013 | Quant - cross-sectional 10 question questionnaire | 1st to 3rd year stds in one university | Turkey | No difference in GSE over three classes. Males > females. No correlation between exam scores and GSE. | Described as how people cope with unexpected events | General SE measure: how people handle their problems, cope with conflicts, etc. |
| Kim & Jang | Changes in medical students' motivation and self-regulated learning: a preliminary study | International Journal of Medical Education | 2015 | Quant - two-phase longitudinal administration of MSLQ | 1st and 2nd year at 1 university | South Korea | No change in SE over time | I expect to do well in this course' | Not congruent; assessment of outcome expectations, not beliefs about capability |
| Lanken, PN; Novack, DH; Daetwyler, C; Gallop, R; Landis, JR; Lapin, J; Subramaniam, GA; Schindler, BA | Use of online clinical videos for clinical skills training for medical students: benefits and challenges. | Academic medicine : journal of the Association of American Medical Colleges | 2015 | Quant - RCT using self-assessed pre- and post surveys (Likert scales) | 2nd and 3rd year at 1 university | USA | Four scales related to self-efficacy, and all of the changes were in the positive direction with small-to-large effect sizes. | How confident are you in your knowledge of substance abuse disorders?' | Congruent |
| Laschinger, HK; Tresolini, CP | An exploratory study of nursing and medical students health promotion counselling self-efficacy. | Nurse Education Today | 1999 | Quant - Cross-sectional study comparing medical and nursing students | 4th year in 2 unis | USA | Self-efficacy scores were high. Nursing students were most confident in their knowledge for counselling clients about nutrition; medical students were most confident about their knowledge for counselling clients about STD prevention. Nursing students were least self-efficacious in their knowledge and ability to counsel patients about smoking cessation; medical students were least self- efficacious in their knowledge and ability to counsel patients about nutrition. | Indicate how confident you are in your ability to counsel patients with regard to that topic. E.g. The health risks related to smoking' | Congruent |
| Lathia, A; Rothberg, M; Heflin, M; Nottingham, K; Messinger-Rapport, B | Effect of a Novel Interdisciplinary Teaching Program in the Care-continuum on Medical Student Knowledge and Self-Efficacy. | Journal of the American Medical Directors Association | 2015 | Quant - Pre-post design with intervention (MedTEC) and control curricula. | 3rd year 1 university | USA | For both the MedTEC and comparison groups, there was a statistically significant increase in scores regarding their attitudes and self-efficacy beliefs about providing care for older adults in different care settings | I am confident in my ability to provide appropriate care for older adults in the hospital' | Congruent |
| Lofaso, D. P; DeBlieux, P. M; DiCarlo, R. P; Hilton, C; Yang, T; Chauvin, S. W | Design and Effectiveness of a Required Pre-Clinical Simulation-based Curriculum for Fundamental Clinical Skills and Procedures | Medical Education Online | 2011 | Quant - pre-post design with clinical skills training. | Year 1 and 2 stds in one university | USA | Students' SE increase after completing clinical skill lab training | Confidence ratings for performing clinical skills | Congruent (but not very clearly described) |
| Lyon, PM; McLean, R; Hyde, S; Hendry, G | Students' perceptions of clinical attachments across rural and metropolitan settings | Assessment & Evaluation in Higher Education | 2008 | Quant - cross-sectional questionnaire was designed to capture the issues students had chosen to focus on in their account of what helps them to learn, using the common words and phrases they had used to describe their experiences. | Year 3 at 1 university | Australia | The items in Factor 4 describe the developing sense of confidence and self-efficacy that comes from participation in patient care. | I got plenty of opportunities to develop procedural skills; There was sufficient trust for staff to ask me to help with patient care' | Not future oriented; measures of other constructs |
| Mason and Ellershaw | Assessing undergrad palliative care education: validity and reliability of two scales | Palliative Medicine | 2004 | Quant - validation of SE in palliative care scale | 4th year in one university | UK | Adequate reliability and internal validity with three stable factors | Answer the following questions by placing X on the line between very anxious and very confident in relation to how you think you would feel about….' | SE is operationalized as existing on a continuum between anxiety and confidence |
| Mason and Ellershaw | Preparing for palliative medicine; evaluation of an education programme for fourth year medical undergraduates. | Palliative Medicine | 2008 | Quant - pre- and post-survey of an educational care program using the Self-efficacy in Palliative Care Scale (SEPC) | 4th year in 1 university | UK | Statistically and educationally significant improvements in self-efficacy to practice palliative medicine (self-efficacy) | Answer the following questions by placing X on the line between very anxious and very confident in relation to how you think you would feel about….' | SE is operationalized as existing on a continuum between anxiety and confidence |
| Mason, S. R; Ellershaw | Undergraduate training in palliative medicine: is more necessarily better? | Palliative Medicine | 2010 | Quant- pre- and post-design where SE is assessed on 'self-efficacy in palliative care scale'. Assessed: communication, patient management, and multiprofessional team work | Year 4 stds in 1 university | UK | Palliative curric intervention increased SE | Answer the following questions by placing X on the line between very anxious and very confident in relation to how you think you would feel about….' | SE is operationalized as existing on a continuum between anxiety and confidence |
| Mavis, Brian | Self-Efficacy and OSCE Performance Among Second Year Medical Students. | Advances in Health Sciences Education | 2001 | Quant - SE questionnaire administered before OSCE; 6 point rating scale | 2nd year in 1 med school | USA | SE was not sig correlated with OSCE performance r = .12; SE neg correlated with anxiety. Stds felt more prepared with higher SE, but didn't score higher | Rate your confidence in your ability to conduct an interview…' | Congruent |
| Metcalf, M. P; Tanner, T. B; Buchanan, A | Effectiveness of an online curriculum for medical students on genetics, genetic testing and counseling | Medical Education Online | 2010 | Quant - SE measured using retrospective pre/post-design questions, asking students to rate responses on a 5 point Likert scale | Online students from 8 medical schools | USA | Self-efficacy increased after web-based course | I am/was confident I can determine appropriate risk' (SE was rated after the performance test) | Congruent |
| Morton, J; Anderson, L; Frame, F; Moyes, J; Cameron, H | Back to the future: teaching medical students clinical procedures | Medical Teacher | 2006 | Quant- questionnaires assessed SE just before assessment of four key procedures | 5th year from one university | UK | Only a weak relationship between observed competence and SE | How confident are you to perform the task (e.g., venepuncture)?' | Congruent |
| Nagoshi, MH; Tanabe, MKG; Sakai, DH; Masaki, KH; Kasuya, RT; Blanchette, PL | The impact of curricular changes on the geriatrics knowledge, attitudes and skills of medical students. | Gerontology and Geriatrics Education | 2008 | Quant - Cross-sectional study of SE, attitudes, and skills before and after curricular changes | All years one university | USA | Students’ self-efficacy increased with curriculum changes (but not statistically significant) | I am well prepared to care for older adult patients in acute settings' and 'Geriatrics education was part of all four years of my medical education' | Not future oriented; measures of other constructs |
| Nieman, LZ; Cheng, L; Foxhall, LE | Teaching first-year medical students to apply evidence-based practices to patient care. | Family Medicine | 2009 | Quant - Three-wave assessment of SE. The student self‐efficacy questionnaire was administered before and after the orientation and after the end of the preceptorship. | Year 1 and 2 stds in one university | USA | The students’ self-efficacy in their ability to provide EBM techniques to patients in their preceptorship increased from 22.2% before orientation to 80.4% after orientation. By the end of the preceptorship, the students’ self-efficacy in using EBM with their patients had decreased to 57.1% | How confident are you that you can use an evidence-based approach to focus your intera tions with your preceptor about specific patients?' | Congruent |
| Ockene, JK; Hayes, RB; Churchill, LC; Crawford, SL; Jolicoeur, DG; Murray, DM; Shoben, AB; et al. | Teaching Medical Students to Help Patients Quit Smoking: Outcomes of a 10-School Randomized Controlled Trial. | Journal of General Internal Medicine | 2016 | Quant - RCT using pre and post test surveys | Year 2 and 4 at 10 unis | USA | Multi-modal tobacco treatment education students were more likely to report higher self-efficacy skills for many 5A and pharmacotherapy counseling tobacco dependence behaviors. | Self-reported skill level for multiple performance tasks | Not congruent; not future oriented, not focused on beliefs of capabilities |
| Papinczak, T | Are deep strategic learners better suited to PBL? A preliminary study. | Advances in Health Sciences Education | 2009 | Mixed methods qual and quant - 11 questions, written by the researcher, dealing with self-efficacy, relating to regulation of, and confidence in, learning. Responses were scored on an ordinal Likert scale | 1st year in one university | Australia | Deep strategic learners had strong self-efficacy and a preference for courses supporting development of understanding | I am able to complete my allocated learning objectives for my tutorial group on time' | Not future oriented; assesses current knowledge, not capability to carry out a course of action |
| Papinczak, T; Young, L.; Groves, M; Haynes, M | Effects of a Metacognitive Intervention on Students' Approaches to Learning and Self-Efficacy in a First Year Medical Course | Advances in Health Sciences Education | 2008 | Quant - pre- and post-test questionnaire with intervention and control groups, Likert scale questions | 1st year in one university | Australia | SE scores declined over the year. No diff between SE in intervention and control groups. | Self-efficacy for self-regulation and five items self-efficacy for academic achievement (adapted from Schwarzer but focused on specific domains) | Congruent |
| Papinczak, T; Young, L; Groves, M; Haynes, M | An analysis of peer, self, and tutor assessment in problem-based learning tutorials. | Medical Teacher | 2007 | Mixed methods qual and quant - The instrument to measure students’ self-efficacy was composed specifically for this project as existing instruments were not designed for use in problem-based learning courses. The test of self-efficacy comprises eleven closed questions relating to regulation of, and confidence in, learning, with scores rated on a Likert scale of one-to-five. | 1st year in one university | Australia | Self-efficacy was correlated with self-assessment. Students awarding themselves higher marks were more likely to have stronger self-efficacy for self-regulation. | Self-efficacy for self-regulation and five items self-efficacy for academic achievement (adapted from Schwarzer but focused on specific domains) | Congruent |
| Pelaccia, T; Delplancq, H; Triby, E; Bartier, J.-C; Leman, C; Dupeyron, J.-P. | Impact of training periods in the emergency department on the motivation of health care students to learn. | Medical Education | 2009 | Quant - At the beginning of the course, the students completed an anonymous 26-item questionnaire to assess their motivational orientations. | Different year/different unis | France | Positive correlation between self-efficacy perception and perceived task value. Prior confrontation with negative outcome events was associated with a negative impact on students’ EFF for learning and achieving in emergency medicine. | I am confident I will understand the emergency medicine material in this course' | Congruent |
| Pelaccia, T; Delplanq, H; Triby, E; Bartier, J-C; Leman, C; Hadef, H; Pottecher, T; Dupeyron, J-P | Gender Stereotypes: An Explanation to the Underrepresentation of Women in Emergency Medicine | Society for Academic Emergency Medicine | 2010 | Quant - cross-sectional SE for practicing emergency medicine | 3rd year from three unis | France | SE was negatively correlated with female students' belief that emergency medicine careers are better suited for men | I am confident I will understand the emergency medicine material in this course' | Congruent |
| Pololi & Price | Validation and use of an instrument to measure learning environment | medical education | 2009 | Quant - scale validation with survey administered to four medical school classes | all 4 years of one university | USA | SE diminishes over 4 years (cross-sectional) | I believe I will learn to be an effective doctor; and 'I believe my fellow stds respect me' | Does not consistently measure beliefs about capabilities; is not future oriented |
| Pololi, L; Potter, S; Garber, CE | A competency-based preventive medicine teaching module for medical students. | Teaching and Learning in Medicine | 1998 | Quant - pre- and post-test survey administered before a course on emergency medicine Measured at the beginning and end of the clerkship | 3rd year 1 university | USA | Significantly increased levels of student self-efficacy for both risk factor screening and fostering behavioral change in patients | How confident are you about your ability to… e.g. Screen for and diagnose alcohol abuse' | Congruent |
| Ruiz, JG; Smith, M; Rodriguez, O; van Zuilen, MH; Mintzer, MJ | An interactive e-learning tutorial for medical students on how to conduct the performance-oriented mobility assessment. | Gerontology and Geriatrics Education | 2007 | Quant - pre and post test questionnaires using a SE scale to measure performance-oriented mobility assessment | 2nd year in one university | USA | Students who accessed the tutorial showed significant increases in skill self-efficacy, and there was a modest correlation between post-tutorial self-efficacy ratings and quiz scores. | Not given | Not possible to evaluate |
| Saketkoo, L; Anderson, D; Rice, J; Rogan, A; Lazarus, CJ | Effects of a disability awareness and skills training workshop on senior medical students as assessed with self ratings and performance on a standardized patient case. | Teaching and Learning in Medicine | 2004 | Quant - `pre- and post-test self-efficacy questionnaire before and after clerkship | 4th year at 1 university | USA | The treatment group changed more than the control group on the Advocacy scale | People with disabilities can be effective parents; I recognize the impact of disabilities on people's lives' | Not measuring beliefs about capabilities; not future-oriented |
| Schauber, SK; Hecht, M; Nouns, ZM; Kuhlmey, A; Dettmer, S | The role of environmental and individual characteristics in the development of student achievement: A comparison between a traditional and a problem-based-learning curriculum | Advances in Health Sciences Education | 2015 | Quant - Longitudinal study assessing SE of students in traditional and PBL programs | Between the 6th and the 10th semester at one university (but two separate curriculums) | Germany | Social support was related to general self-efficacy and student perception of the learning environment. GSE was also positively related to postive study-related affect. | I can always manage to solve difficult problems if I try hard enough | Not domain specific; general self-efficacy |
| Stegers-Jager, K. M; Cohen-Schotanus, J; Themmen, A. P. N | Motivation, learning strategies, participation and medical school performance | Medical Education | 2012 | Quant - cross-sectional questionnaire 2 months after enrolling in medical school with Year 1 performance as outcome | 1st year in one university | Netherlands | SE correlated with Year 1 grade (and was not mediated by learning strategies). | I expect to do well in this course' | Not congruent; assessment of outcome expectations, not beliefs about capability |
| Sullivan, M; Nyquist, J; Etcheverry, J; Nally, M; Schaff, P; Abbott, A; Elliott, D; Taylor, C | The development of a comprehensive school-wide simulation-based procedural skills curriculum for medical students. | Journal of Surgical Education | 2010 | Quant - Student self-confidence was evaluated using a retrospective pre-post-self confidence rating for each skill | 2nd year at 1 university | USA | Student self-efficacy regarding each skill increased significantly after the workshops | Self-confidence rating for each skill | Congruent |
| Tresolini, C. P; Stritter, F. T | An analysis of learning experiences contributing to medical students' self-efficacy in conducting patient education for health promotion | Teaching and Learning in Medicine: An International Journal | 1994 | Qual with some quant - case-study approach, data gathered through student and faculty interviews, document review and questionnaire | 4th year in one university | USA | SE for patient educ were very high. SE increased with learning experiences. | How confident are you that you can convey to your patients the information they need to quit smoking?' | Congruent |
| Turan, S; Konan, A | Self-Regulated Learning Strategies Used in Surgical Clerkship and the Relationship with Clinical Achievement | Journal of Surgical Education | 2012 | Quant - test of predictive validity between SE and OSCE scores | 4th year in 1 university | Turkey | SE was related to score in surgery and with OSCE but not with case-based exam score or tutor score | I expect to do well in this course' | Not congruent; assessment of outcome expectations, not beliefs about capability |
| van de Ridder, JMM; Peters, CMM; Stokking, KM; de Ru, JA; ten Cate, OTJ | Framing of feedback impacts student's satisfaction, self-efficacy and performance | Advances in Health Sciences Education | 2015 | Quant - RCT where students were asked to rate self-efficacy after: 1) being asked about procedure 2) watching intructional video, and performing procedure, 3) receiving +/- feedback and performing procedure again, 4) two weeks after | 1st year in one university | Holland | The linear mixed model showed an interaction effect between self-efficacy and time. Feedback from the task affected the students' feeling of competence regarding the task, which can explain further increase of self-efficacy in the positively framed feedback condition and decrease in the negatively framed feedback condition. | Visual analogue scale (from 'extremely confident' to 'extremely uncofident') to detect hearing loss | Congruent |
| Van Nguyen, H; Laohasiriwong, W; Saengsuwan, J; Thinkhamrop, B; Wright, P | The relationships between the use of self-regulated learning strategies and depression among medical students: an accelerated prospective cohort study. | Psychology, health & medicine | 2015 | Quant - Prospective cohort study with data collected at two time points | 1st to 5th years at one university | Vietnam | Self-efficacy was a significant predictor of depression | I expect to do well in this course' | Not congruent; assessment of outcome expectations, not beliefs about capability |
| Vincent, DS; Sherstyuk, A; Burgess, L; Connolly, KK | Teaching mass casualty triage skills using immersive three-dimensional virtual reality. | Academic Emergency Medicine | 2008 | Quant - Pre- and post-test self- efficacy questionnaire before and after immersive virtual reality intervention | Multiple years at one university | USA | All five self-efficacy questions showed a statistically significant increase in scores over time. | I feel confident that I will learn to be an effective first responder | Congruent |
| Walter & Kerr | An analysis of third year medical students' knowledge | journal of popular pharmacology | 2011 | Quant - cross-sectional SE questionnaire pertaining to alcohol use in pregnant women | 3rd year from 2 unis | USA | Conseling and screening SE scores low | How confident are you that you can convey to your patients the information they need to quit drinking?' | Congruent |
| Woods, JL; Pasold, TL; Boateng, BA; Hense, DJ | Medical student self-efficacy, knowledge and communication in adolescent medicine. | International journal of medical education | 2014 | Quant - SE scores collected at beginning and end of pediatric rotation | 3rd year 1 university | USA | SE levels increased by the end of the rotation | Ratings of confidence for a range of tasks (interviewing adolescents, asking personal questions) rated on 'not confident' to 'very confident' scale | Congruent |
| Woolf, K; Elton, C; Newport, M | The specialty choices of graduates from Brighton and Sussex Medical School: a longitudinal cohort study. | BMC Medical Education | 2015 | Quant - cross-sectional questionnaire of medical students after choosing speciality | 2 penultimate year groups at 1 university | UK | Respondents with higher self-efficacy were lower on neuroticism, higher on extraversion, and higher on conscientiousness. Self-efficacy was not related to confidence. | Not provided, but used GSE, e.g., 'I can always manage to solve difficult problems if I try hard enough' | Not domain specific; general self-efficacy |
| Xiao, R. S., et al. | Tobacco counseling experience prior to starting medical school, tobacco treatement self-efficacy and knowledge among first-year medical students in the United States | Preventive Medicine | 2015 | Quant - cross-sectional survey looking at tobacco counseling self-efficacy | 1st year med stds from 10 unis | USA | Students with prior counseling experiences were more likely to have higher tobacco counseling self-efficacy | How skilled are you in tobacco counseling skills? | Not future-oriented; not focus on beliefs of capabilities |
| Young, H. N; Schumacher, J. B; Moreno, M.A; Brown, R. L; Sigrest, T. D; McIntosh, G. K; Schumacher, D. J; Kelly, M. M; Cox, E. D | Medical Student Self-Efficacy With Family-Centered Care During Bedside Rounds | Academic Medicine | 2012 | Quant - cross-sectional SE measurement with self-reported self-efficacy measured on 11 items in a survey on 7-point Likert-type scale | 3rd years from 1 health centre | USA | Feedback didn't influence SE. Observing role models and practicing for mastery fosters SE. Implications for SOSE | I can build trust with patients and families during family-centered bedside rounds | Congruent |
| Zachariae, R; O'Connor, M; Lassesen, B; Olesen, M; Kjaer, LB; Thygesen, M; Morcke, AM | The self-efficacy in patient-centeredness questionnaire - a new measure of medical student and physician confidence in exhibiting patient-centered behaviors | BMC Medical Education | 2015 | Quant - development of scale to assess self-efficacy in patient-centeredness | Medical students at 1 university | Denmark | Results indicate that the SEPCQ-27 is a reliable and valid instrument for assessing patient- centeredness self-efficacy in both medical students and physicians | I am confident that I am able to ... e.g., Make the patient feel that I have time to listen' | Congruent |
